# Bioinspired Cilia Sensors with Graphene Sensing Elements Fabricated Using 3D Printing and Casting

**DOI:** 10.3390/nano9070954

**Published:** 2019-06-30

**Authors:** Amar M. Kamat, Yutao Pei, Ajay G.P. Kottapalli

**Affiliations:** 1 Advanced Production Engineering Group, Engineering and Technology Institute Groningen, Faculty of Science and Engineering, University of Groningen, Nijenborgh 4, 9747AG Groningen, The Netherlands; 2 MIT Sea Grant College Program, Massachusetts Institute of Technology, 77 Massachusetts Avenue, Cambridge, MA 02139, USA

**Keywords:** 3D printing, biomimetic sensor, flexible electronics, graphene, PDMS, gauge factor

## Abstract

Sensor designs found in nature are optimal due to their evolution over millions of years, making them well-suited for sensing applications. However, replicating these complex, three-dimensional (3D), biomimetic designs in artificial and flexible sensors using conventional techniques such as lithography is challenging. In this paper, we introduce a new processing paradigm for the simplified fabrication of flexible sensors featuring complex and bioinspired structures. The proposed fabrication workflow entailed 3D-printing a metallic mold with complex and intricate 3D features such as a micropillar and a microchannel, casting polydimethylsiloxane (PDMS) inside the mold to obtain the desired structure, and drop-casting piezoresistive graphene nanoplatelets into the predesigned microchannel to form a flexible strain gauge. The graphene-on-PDMS strain gauge showed a high gauge factor of 37 as measured via cyclical tension-compression tests. The processing workflow was used to fabricate a flow sensor inspired by hair-like ‘cilia’ sensors found in nature, which comprised a cilia-inspired pillar and a cantilever with a microchannel that housed the graphene strain gauge. The sensor showed good sensitivity against both tactile and water flow stimuli, with detection thresholds as low as 12 µm in the former and 58 mm/s in the latter, demonstrating the feasibility of our method in developing flexible flow sensors.

## 1. Introduction

Biological sensors found in living beings ranging from bacteria to plants to mammals display sensing capabilities that are unrivalled by any comparable man-made technologies, both in sensitivity and versatility, owing to millions of years of optimization through evolution and natural selection. The creative micromechanical designs of various biological sensors such as acoustic sensors in the inner ear, olfactory sensors in sharks, neuromast flow sensors in fishes, wake sensing whiskers in seals, tactile sensors in human finger tips, thermal sensors in beetles, and so on, exhibit impressive sensitivity and high efficiency in filtering biologically-relevant signals in noisy ambient conditions [1,2,3]. In the pursuit of efficient microelectromechanical systems (MEMS) sensors, researchers have taken inspiration from natural sensors mainly by mimicking their unique morphology, materials, geometry, and functionality [4,5,6,7,8,9].

**Figure 1:**
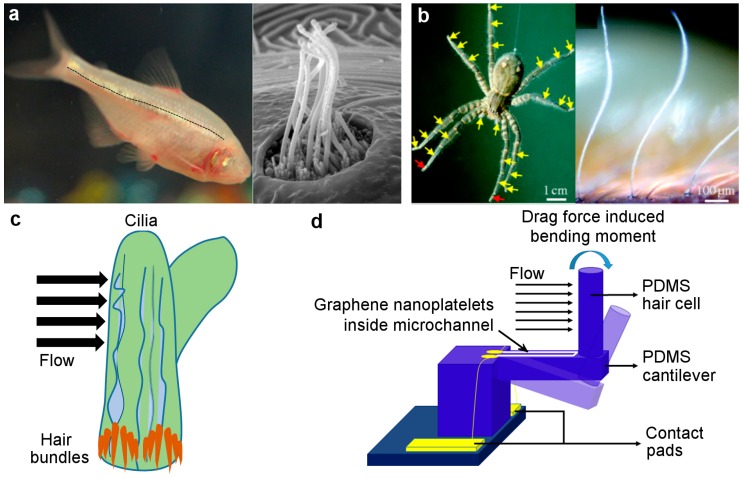
Biomimetic flow sensing: (**a**) lateral line sensors on fish skin (shown by black dotted line) containing hair-like cilia bundles (credit: Prof. Andrew Forge [20]) for water flow sensing; (**b**) hair-like sensilla on spider legs (reprinted with permission from [14], Copyright The Royal Society, 2008); (**c**) schematic of flow-induced bending of cilia bundles encapsulated by a protective cupula; and (**d**) sensing principle of bioinspired sensor comprising hair cell and cantilever used in this work.

Ultrasensitive hair-like ‘cilia’ structures are ubiquitous in nature and perform flow sensing in numerous animal species, examples of which are shown in Figure 1a (blind cavefish or *Astyanax mexicanus fasciatus*) and Figure 1b (tiger wandering spider or *Cupiennius salei*). The sensing principle of the cilia in all these species is similar, namely, that the drag force induced by the flow is translated to mechanical bending of the high-aspect ratio cilia, which in turn elicits an electrical impulse across the hair cell membrane located at the base of the cilia (Figure 1c). Cilia sensors in crickets are capable of detecting airflow velocities as low as 0.05 mm/s [10], while the neuromast cilia sensors in fishes can detect steady-state water flow velocities down to 10 mm/s [11] and oscillatory flow velocities as low as 10–38 µm/s in the 10–20 Hz range [12,13]. The hair-like sensilla on spider legs are sensitive to air flow perturbation energies as low as 2.5 × 10^−20^ Joules [14]. Mechanosensitive ‘stereocilia’ bundles achieve ultrafast and sub-Brownian threshold detection of sound, linear acceleration, and angular velocity by exhibiting microsecond response times and nanometer-scale deflection sensitivities in the inner ear of mammals [15]. Similar to the cilia, seals use their whiskers as ultrasensitive flow sensors to detect wake trails generated by fishes, allowing them to hunt prey swimming 180 meters away [16]. The dimensions of most biological cilia range from 2–1500 µm in height and 0.2–500 µm in diameter, thus allowing cilia-inspired sensors to be fabricated using MEMS technology [17]. Researchers have achieved impressive sensitivity (as high as 0.077 V/m s^−1^) and threshold velocity detection limits (as low as 0.015 m s^−1^) by closely mimicking, for instance, the anatomy and functionality of lateral line sensors found in fishes [18,19].

Artificial MEMS cilia sensors have been developed in the past using conventional micro/nano fabrication processes utilizing either silicon or SU-8 polymer as the structural material embedded with piezoelectric or piezoresistive sensing elements. While some researchers used hot wire anemometry [21], capacitive sensing [22], and optical sensing [23] to develop flow sensors inspired by cilia, others focused on mimicking the drag-force induced bending of the cilia which followed the sensing principle of the biological cilia and allowed utilization of biomimetic material-induced sensitivity enhancement of the sensor [18,24,25,26,27]. Inspired by flow sensing cilia in crickets, Casas et al. [28] developed MEMS flow sensors featuring 825 µm tall SU-8 polymer cilia with reduced diameter at the distal tip through double layer polymer deposition and lithographic patterning. Yang et al. [21] developed an array of three-dimensional (3D), out-of-plane, MEMS flow sensors that used hot wire anemometry to detect flows and perform distant touch hydrodynamic imaging similar to the neuromast sensors in fishes. The 3D sensing element was fabricated through surface micromachining to form a nickel-iron alloy support prong and a nickel-polymer composite hot-wire, after which the surface micromachined planar device was converted into a 3D structure through a magnetically assisted assembly step that rotated the cantilevers out-of-plane and spatially elevated the hot-wire sensing element [21]. Chen et al. [27] developed cilia-inspired MEMS flow sensors featuring a SU-8 resist polymer cylinder, 600 µm tall and 80 µm in dimeter, positioned at the distal tip of a 2 µm thick and 20 µm wide silicon double cantilever beam structure embedded with ion-implanted piezoresistors at the hinge. Similar to the biological cilia sensors, these MEMS cilia transduced the drag force-induced bending to the sensing element at the base. These sensors were capable of sensing steady-state and oscillatory water flow velocities as low as 25 mm/s and 1 mm/s, respectively, and could achieve an angular flow direction resolution of 2.16° in air flow [27]. Alfadhel et al. [29] developed cilia-inspired tactile sensors out of PDMS and iron nanowires; the magnetic sensing element was fabricated using standard lithography procedures while the cilia was cast out of a master mold with laser-drilled holes. Kottapalli et al. [18,25] developed an all-polymer, cilia-inspired, MEMS flow sensor which featured 3D-printed (via stereolithography) polycarbonate cilia positioned on a liquid crystal polymer sensing membrane deposited with serpentine shaped, photo-patterned, gold strain gauges [12,13]. These sensors were mainly used to sense steady-state flows and to demonstrate ‘touch at a distance’ underwater object imaging. Asadnia et al. [24] used the same polycarbonate cilia but on a lead zirconium titanate (PZT, Pb[Zr_0.52_Ti_0.48_]O_3_) piezoelectric membrane to form self-powered cilia flow sensors that successfully detected near-field dipole stimuli in both air and water. 

Most of the biomimetic cilia-inspired MEMS flow sensors discussed above utilized conventional microfabrication techniques which were cumbersome and involved multilayer deposition and lithography steps, especially when fabricating high-aspect ratio cilia structures. Moreover, they were limited by the number of materials (usually silicon or SU-8 polymer) that could be used in the fabrication, making them unsuitable for flexible sensing applications. To truly mimic the ultrasensitivity of naturally-occurring cilia and implement it in artificial flow sensors, a combination of a soft polymer pillar structure (allowing high bending strains) and high-gauge factor strain sensing materials is essential. Several nanomaterials such as silver nanowires [30] and nanoparticles [31,32], zinc oxide nanowires [33], carbon black [34], carbon nanotubes [35], graphene oxide [36,37], and graphene [38,39,40,41,42,43,44] have shown promise when used as flexible strain sensors, where the nanomaterials were either mixed with the soft polymer to form a nanocomposite or were deposited as a thin film on a flexible substrate [45,46]. In particular, the use of graphene as a piezoresistive strain sensor has been actively explored in the literature due to the high gauge factors achievable [47]. The unique two-dimensional (2D) structure of graphene facilitates easy sliding between neighboring flakes, causing a large change in contact area (and hence contact resistance) upon the application of strain [44,48] leading to higher gauge factor values than conventional piezoresistive strain gauges.

3D-printing technology has recently emerged as a promising technique for rapid manufacturing of sensors [49,50,51], but its application towards fabricating bioinspired, flexible, MEMS flow sensors has been limited, since direct printing of soft polymers (such as polydimethylsiloxane) is challenging owing to their low Young’s modulus values and long curing times. Although some researchers [18,24,52] used a hybrid approach by mounting 3D-printed cilia structures manually on photolithographically fabricated sensing membranes, monolithic fabrication and integration of cilia-inspired, 3D MEMS structures and sensing elements remains a major challenge. 

In this work, we designed and fabricated a cilia-inspired flow sensor using polydimethylsiloxane (PDMS) for the sensor structure and graphene nanoplatelets (GN) as the piezoresistive sensing elements. The 3D sensor structure in this work was fabricated by casting PDMS into a 3D-printed, stainless steel mold. The bioinspired sensor design comprised an all-PDMS cantilever-pillar structure with a GN piezoresistor deposited on the cantilever surface (Figure 1d). The drag force-induced bending of the pillar, and thereby the cantilever, due to flow was sensed by a change in resistance of the piezoresistive sensing elements (i.e., GN) located inside the microchannel. Uniaxial tension-compression tests were conducted to characterize the graphene-on-PDMS strain gauge. Oscillatory and steady-state tests were performed to gauge the sensitivity of the cilium sensor for both flow and touch stimuli. The original aspects of our work include: i) a novel, low-cost, and repeatable processing technique, involving a combination of high-resolution metal 3D printing and polymer casting, to fabricate flexible and 3D sensor structures with intricate features; ii) the use of GN as a piezoresistive sensing element for flow sensing; and iii) the creation of high sensitivity in bioinspired MEMS flow sensors using a combination of flexible sensor structures and high-gauge factor graphene sensing elements. The fabrication methods described in this work alleviate the cumbersome and expensive multilayer deposition and lithography steps required to fabricate complex 3D structures (e.g. high-aspect ratio pillars) and/or intricate features (e.g. microchannels). The proposed methodology also allows the possibility of using a wide range of polymer materials for MEMS fabrication. Finally, the 3D printing and casting approach described in this work can potentially pave the way to the development of other biomimetic 3D-printed sensor structures in the future.

## 2. Materials and Methods 

### 2.1. Sensor Fabrication

#### 2.1.1. 3D Printing of the Metallic Mold

The flow sensor structure utilized a cantilever-pillar design, with a vertically standing cylindrical hair-like structure (Ø 1.5 mm × 4 mm) located at the free end of the horizontal cantilever (4.5 mm × 1.5 mm × 1.5 mm); further, a U-shaped microchannel (0.3 mm × 0.3 mm × 6.5 mm) designed on the top surface of the cantilever accommodated the GN sensing element. The metallic mold for the sensor, i.e. the ‘negative’ of the sensor design, was 3D-printed using the selective laser melting process (SLM 125^HL^, SLM Solutions GmbH, Lübeck, Germany) [53], where a focused laser beam selectively fused 17–4 PH stainless steel powder (10-50 µm size distribution, LPW Carpenter Additive, Runcorn, UK) into the final 3D mold shape in a layer-by-layer manner, using a powder layer thickness of 30 µm. The focused laser beam had a spot size of 70 µm, making the minimum feature size printable with this process to be 140 µm in the build plane. Manufacturer-recommended processing parameters (e.g. 200 W laser power, 800 mm/s laser scan speed, 120 µm hatch spacing, etc.), optimized to maximize the density of the 3D-printed mold, were used for the selective laser melting (SLM) process. After printing, the inner walls of the 3D-printed mold were first sandblasted to improve their surface quality and then lubricated using a commercially available degreaser (WD-40, San Diego, CA, USA) to facilitate demolding.

#### 2.1.2. PDMS Casting and GN Infusion

**Figure 2:**
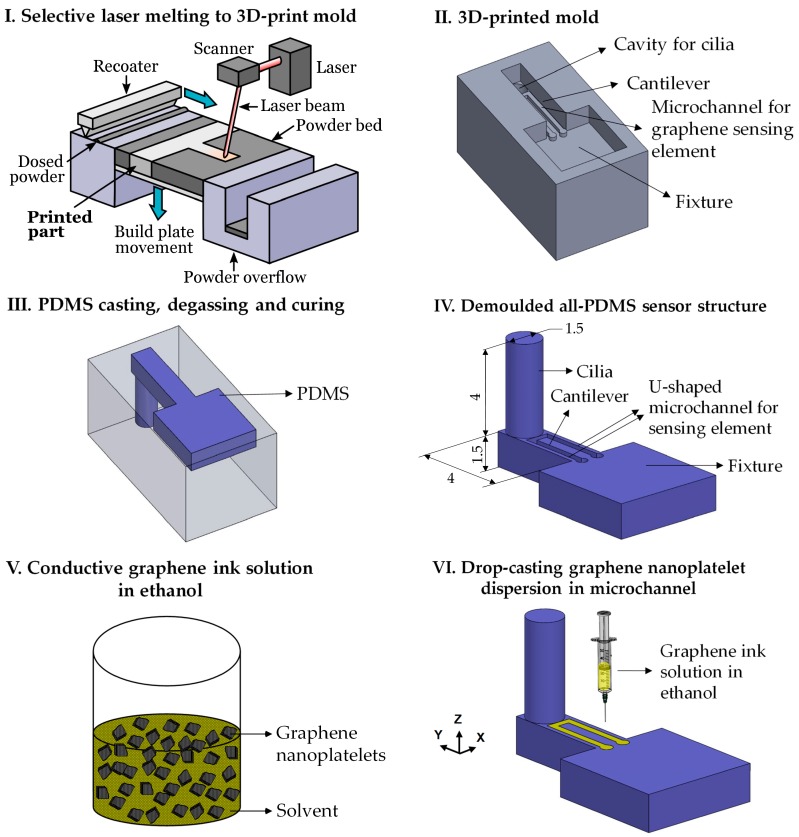
Schematic of fabrication process flow involving metal 3D printing (selective laser melting), PDMS casting, and graphene infusion into microchannel. Selective laser melting (SLM) process schematic (Image I) reprinted with permission from [54], Copyright Elsevier, 2019. All dimensions in Image IV are in mm. The size of the graphene nanoplatelets in Image V is exaggerated.

**Figure 3:**
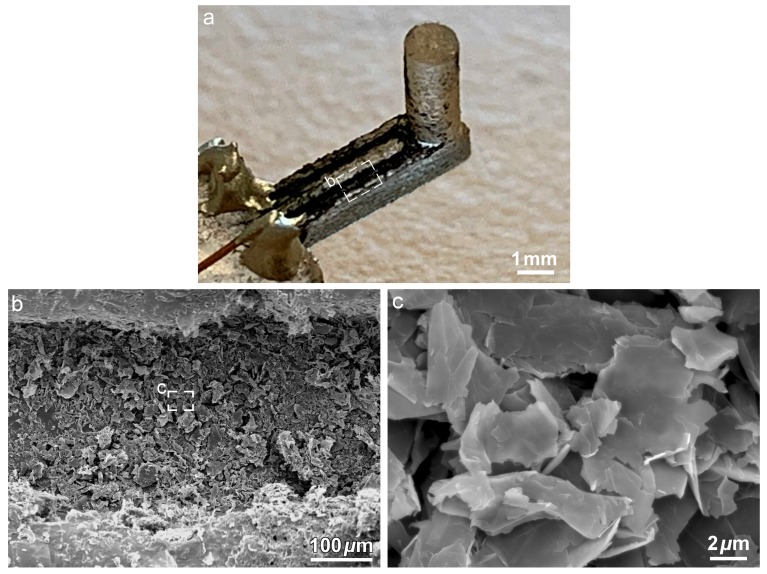
Micrographs of sensor structure with graphene infused into the microchannel: (**a**) optical micrograph of the developed sensor, (**b**) SEM image of GN inside the microchannel and (**c**) high magnification SEM image showing the morphology of GN sensing elements.

PDMS (SYLGARD ^TM^ 184, Dow Corning, Midland, MI, USA) solution was prepared by thoroughly mixing a ratio of 10 parts base monomer to 1 part curing agent by weight, after which it was degassed in a vacuum chamber for 40 min, poured into the 3D-printed mold, degassed for another 10 min, allowed to cure at room temperature for a period of 48 h, and finally demolded to obtain the sensor structure. Conductive graphene dispersion (Graphene Supermarket, Ronkonkoma, NY, USA), consisting of a solution of GN (average thickness of 7 nm) in n-butyl acetate and a proprietary dispersant (23 wt. % graphene), was further diluted with ethanol to reduce its viscosity and then drop cast into the microchannel on the cantilever surface using a syringe and a 22-gauge needle. The GN solution flowed easily in the microchannel due to the capillary effect and formed a thin film on the PDMS substrate upon drying, after which it was gently annealed at 120 °C for 30 minutes to improve its conductivity as per the supplier’s recommendation. Electrical connections were made at the ends of the microchannel using conductive silver paste (EPOTEK H20E, Epoxy Technology Inc., Billerica, MA, USA). A schematic of the sensor fabrication work flow is presented in Figure 2. Figure 3a shows an optical micrograph of the sensor structure with graphene infused into the microchannel, while Figure 3b,c show scanning electron microscopy (SEM) micrographs of the GN inside the microchannel, demonstrating that the GN were successfully drop-cast into the microchannel without any unintended connections across it. The GN sensing elements were homogeneously distributed inside the microchannel and contacted each other.

### 2.2. Gauge Factor Characterization

Since the GN strain gauge present on the top surface of the cantilever forms the fundamental piezoresistive sensing element, the determination of its gauge factor (GF) is a crucial step towards the sensor characterization. The GF of a strain gauge, defined as the ratio of the fractional resistance change ($\frac{R-R_{0}}{R_{0}}=\frac{\Delta R}{R_{0}}$) to the mechanical strain (ɛ), where *R* and *R_0_* are the sensor resistances in the stressed and unstressed conditions respectively, was measured through a uniaxial tension-compression test. A rectangular cuboid tensile test specimen (50 mm × 10 mm × 10 mm) with a microchannel (23 mm × 0.3 mm × 0.3 mm) on one surface was cast in PDMS using a 3D-printed mold, after which GN was drop-cast into the microchannel and gently annealed using the procedure described in Figure 2. Electrical connections were made at the ends of the microchannel using conductive silver paste. The rectangular tensile specimen was subjected to 10 tensile-compressive cycles using a micromechanical testing machine (Kammrath & Weiss GmbH, Dortmund, Germany). 10 mm of the tensile specimen length was clamped on each side during the test, giving a gauge length of 30 mm which was used for all the strain calculations. Each strain cycle started at a compressive strain of −1.92%, and consisted of: i) ramping up to a tensile strain of 1.92%, ii) holding at the 1.92% tensile strain for 30 seconds, iii) ramping down to a compressive strain of −1.92%, and iv) holding the −1.92% compressive strain for 30 s. A constant strain rate ($\frac{\Delta \varepsilon }{\Delta t}$) of ± 6.67 × 10^-4^ s^−1^ was used for all the ramps, making each cycle approximately 174 s long. The resistance of the sensor was continuously monitored via the setup described in Section 2.3.1.

### 2.3. Sensor Testing

#### 2.3.1. Data Acquisition

The two ends of the GN-containing microchannel were connected to a Wheatstone bridge circuit powered by a 9 Volt direct current (DC) power supply, and the unamplified voltage output from the sensor was continuously monitored using a National Instruments (Austin, TX, USA) data acquisition system (NI-DAQ UBS-6003) and recorded using the NI Signal Express software. The sampling rate for the gauge factor and oscillatory flow sensing experiments were 10 and 1000 Hz, respectively. For the gauge factor characterization, the recorded voltage was converted to electrical resistance using Kirchhoff’s laws applied to the Wheatstone bridge circuit [55].

#### 2.3.2. Experimental Setup for Oscillatory Stimuli

A vibrating dipole apparatus, described in detail in Ref. [24], was used for the oscillatory stimuli. The dipole stimulus was chosen for the flow sensing experiments since the flow field generated by an oscillating sphere is well studied and has been used by other researchers in the past [24,27] for characterizing artificial cilia sensors. In brief, the apparatus consisted of a vibrating stainless steel sphere (8 mm diameter) or ‘dipole’ whose driving voltage, frequency, and oscillatory function (e.g. sinusoidal, saw-tooth, etc.) could be tuned as desired. A permanent magnet mini-shaker (Bruel & Jkaer model 4810, Norcross, GA) having an axial resonant frequency greater than 18 kHz was used to drive the dipole at the desired frequencies and amplitudes. The driving voltage and frequency determined the amplitude and root-mean-square (RMS) velocity of the oscillating sphere; this relationship has been determined in the past through laser doppler vibrometry (LDV) for a sinusoidal driving function [24]; thus, it was possible to independently and accurately vary the frequency and the amplitude/RMS velocity of the vibrating dipole.

## 3. Results

### 3.1. Gauge Factor of Graphene-on-PDMS Strain Gauge

**Figure 4:**
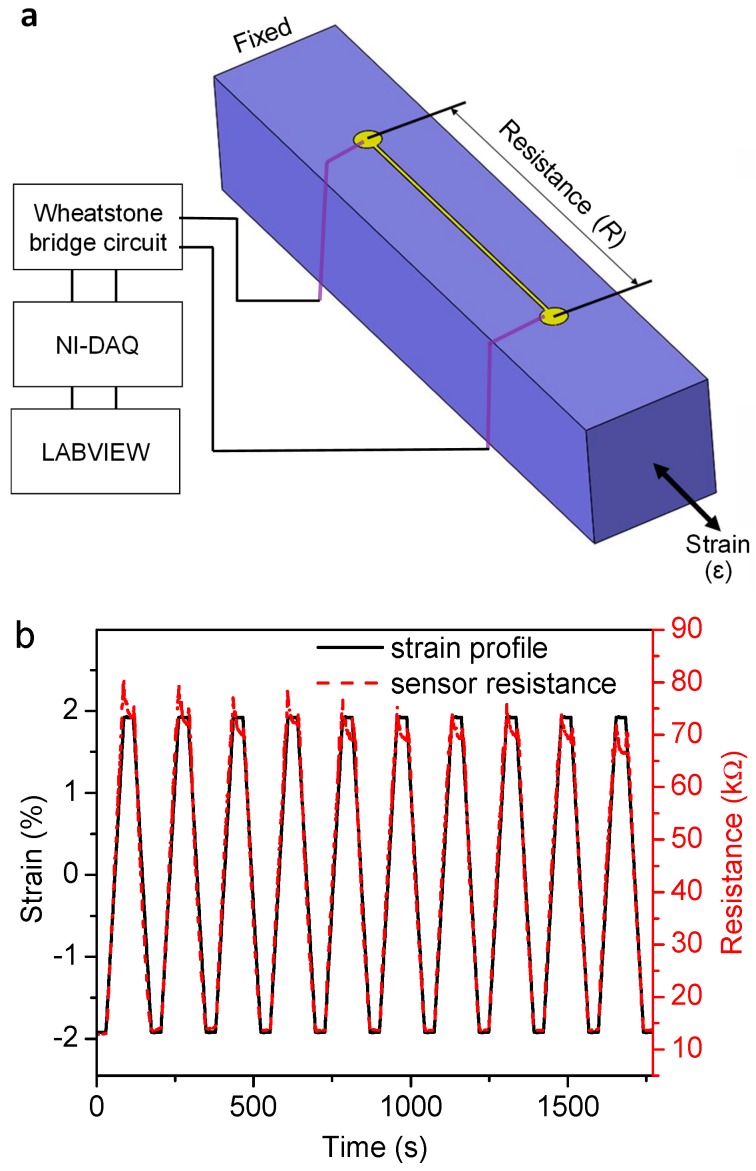
Gauge factor measurement: (**a**) schematic of tensile test setup to measure resistance for an applied strain (blue: PDMS, yellow: graphene); and (**b**) applied strain profile and measured resistance change for 10 tension-compression cycles.

Figure 4 shows the measured resistance of the graphene strain gauge for the applied tensile-compressive strain profile. The resistance change was observed to be linear and nearly identical during both the elongation and compression regions of the ten cycles, with a resistance change rate (Δ*R*/Δ*t*) of 1.135 ± 0.053 kΩ/s calculated by averaging over a total of twenty (ten ramp-up and ten ramp-down) regions. The GN showed excellent recovery over the course of all the ten cycles; the resistance during the compressive hold period was steady, while it drifted by around 5 kΩ during every tensile hold period, indicating higher stability in compression than in tension. Using the strain rate in the linear ramp region (Δɛ/Δ*t* = 8.7 × 10^−4^ s^−1^) and the resistance of the unstressed sample (*R_0_* = 46 kΩ), the average GF was calculated to be 37 ± 1.7, which is in the range of GF’s reported in the literature for graphene strain gauges on elastomeric substrates, as shown in Table 1.

The measured GF for GN is one order of magnitude higher than the GF for a comparable copper strain gauge on a flexible substrate which had a gauge factor of 3 [56]. This can be attributed to the piezoresistivity exhibited by the GN, where the change in resistance due to the applied strain is due not only to geometrical changes (as in metal strain gauges) but rather to a change in resistivity caused by slippage of neighbouring nanoplatelets; tensile strains cause the nanoplatelets to slip away from each other, decreasing the contact area and hence increasing the contact resistance, whereas compressive strains have the opposite effect and reduce the contact resistance [44,48]. The high GF measured in this study thus demonstrated the potential of our methodology for fabricating flexible graphene-on-PDMS strain gauges, and the utility of such a flexible strain gauge in flow and tactile sensing is described in Section 3.2.

### 3.2. Characterization of the Biomimetic Cilium Sensor

**Figure 5:**
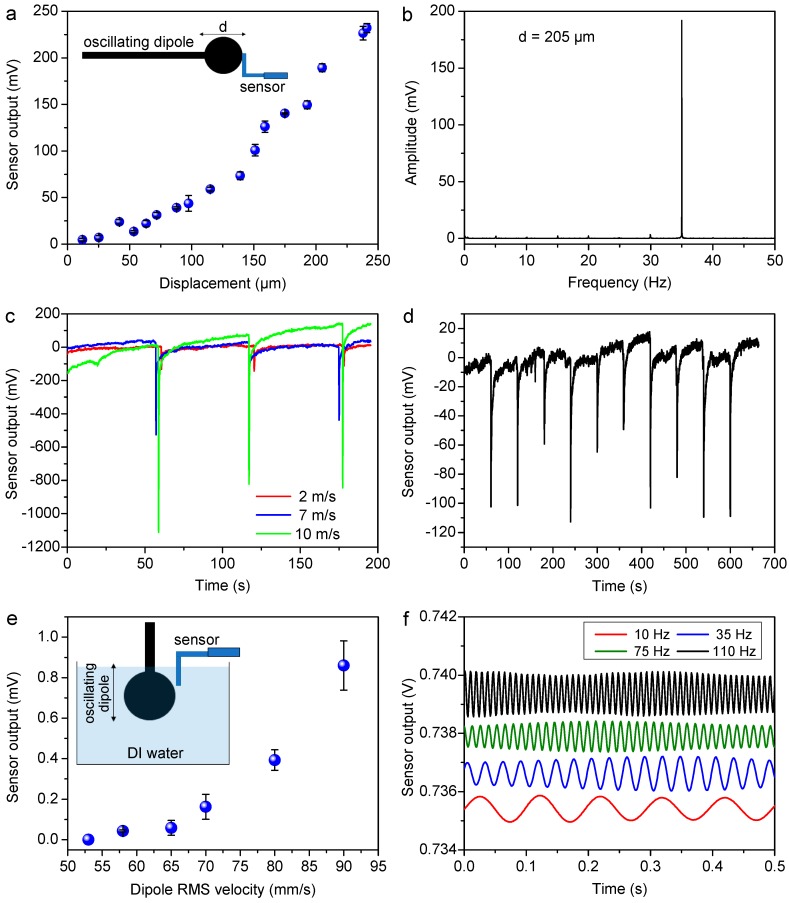
Sensor tests: (**a**) oscillatory tactile stimuli; (**b**) example of FFT peak at 35 Hz for *d* = 205 µm; (**c**) compressed air stimuli along X-axis; (**d**) respiratory exhalation along Y-axis; (**e**) oscillatory flow stimuli in DI water (RMS velocity sweep); (**f**) oscillatory flow stimuli in DI water (frequency sweep).

In order to experimentally characterize the flow sensing performance of the biomimetic flow sensor, a series of static and dynamic flow tests were conducted both in air and water. To understand the relation between the displacement of the cilium and the sensor output, a tactile test was conducted where the cilium was subjected to a known displacement while the voltage output of the sensor was recorded. Since the cilia in nature act not only as flow sensors but also as touch and vibration sensors [29], the tactile sensing performance of the sensor was tested using an oscillatory contact stimulus whose oscillatory amplitude could be accurately controlled. To measure the minimum displacement that could be sensed by the sensor, the dipole was positioned in such a way that its mean position at rest was just touching the hair cell at its tip, and then made to vibrate at a frequency of 35 Hz at different amplitudes ranging from 26 to 241 µm along the Y direction. Each test at a given amplitude was repeated thrice. The voltage-time data for a given test was processed using the Fast Fourier Transform (FFT) operation in the Origin software (OriginLab, Version 2019, Northampton, MA, USA) and the FFT peak (if discernible) at 35 Hz was noted as the sensor output for that particular test and plotted against the oscillatory amplitudes (Figure 5a). The sensor showed a clear peak at the excitation frequency of 35 Hz for all the tested amplitudes (example shown in Figure 5b for *d* = 205 µm). It is evident from Figure 5a that the sensor showed an approximately linear response to the varying amplitude. The maximum strain induced in the cantilever due to a displacement *d* of the cilium tip can be approximated to be [27]:

$$\varepsilon_{max}=\frac{t}{2L}\tan^{-1}\frac{d}{H}=\frac{t}{2L}\frac{d}{H}\;for\;d\ll H$$

where *t* is the cantilever thickness (1.5 mm), *L* is the cantilever length (4.5 mm), and *H* is the cilium height (4 mm), giving a range of 0.1–1% maximum strain in the cantilever for the range of tip displacements applied in the experiment. The relation suggests that the maximum strain in the cantilever (and thus the change in GN resistance) can be assumed to vary linearly with the tip displacement, leading to a linear relationship between the measured sensor output and tip displacement as observed in Figure 5a (average sensitivity ~ 1.02 mV/µm). Further, the sensing threshold, i.e. the lowest tested *d* at which the sensor showed a response, was 12 µm.

The flow sensing performance of the sensor was then characterized for steady flows and oscillatory flows. Figure 5c shows the response of the sensor to a steady-state air flow generated from a flow-controllable air nozzle at velocities of approximately 2, 7, and 10 m/s as measured by a commercial anemometer. The output of the sensor was recorded as the air nozzle was manually swept past the sensor at a distance of 5 mm from the cilium. The direction of sweeping was along the Y-axis whereas the compressed air direction was along the X-axis, according to the coordinate system shown in Figure 2 (image VI). It can be seen from the Figure 5c that increasing the velocity of the air flow showed a corresponding increase of the sensor output due to a greater degree of pillar bending and consequent cantilever torsion. In order to determine the response of the sensor to extremely low steady flow velocities, we recorded the sensor output for respiratory exhalation performed in the vicinity of the sensor approximately 25 mm from the cilium. Figure 5d shows the output of the sensor for air flow exhaled at timed intervals demonstrating possible applications in wearable breathalyzers. The sensor showed very good sensitivity and recovery for both tests, exhibiting its capability of sensing pulsed flows along both the X and Y directions. Moreover, this experiment thus showcased the ability of the graphene strain gauge to exhibit piezoresistivity during both the bending and torsion of the cantilever.

Finally, in order to determine the response of the sensor to dynamic flow stimuli, the sensor output was tested in deionized (DI) water using the vibrating dipole stimulus. In this test, the sphere vibrated along the vertical (i.e. Z) direction with the cilium also oriented along the vertical (–Z) direction. The oscillating sphere was completely submerged in the water, while only the cilium was kept submerged inside the water to avoid contact of the water with the conductive GN strain gauge. The lower tip of the cilium was at a distance 8.48 mm from the center of the vibrating sphere (6mm each along the vertical and horizontal directions), sufficiently far to ensure no contact between the dipole and the cilium at any of the tested amplitudes. Two sweeps were undertaken: varying the RMS velocity (by tuning the RMS driving voltage) of the sphere, from 53 to 90 mm/s at a constant frequency of 35 Hz, and varying the frequency at a constant RMS driving voltage of 707 mV.

In the RMS driving voltage sweep, the FFT of the voltage-time data provided the sensor output at the 35 Hz driving frequency, allowing the sensor output variation as a function of the oscillatory velocity of the dipole. In the frequency sweep, the FFT operation was used to isolate the sinusoidal sensor output at the driving frequency to determine whether the sensor was capable of responding to different frequencies. Figure 5e shows the flow calibration of the sensor for oscillatory flow stimulus in DI water. The sensor demonstrated a sensitivity of 30 mV/(m s^−1^) in the velocity range of 65–90 mm/s which was in the range of reported sensitivities (0.6–44 mV/m s^−1^) for recently reported cilia-inspired flow sensors tested under similar conditions [24,57]. For the RMS velocity sweep at a constant frequency of 35 Hz (Figure 5e), the sensor could not detect the lowest tested RMS velocity of 53 mm/s, thus giving a sensing threshold of 58 mm/s for the RMS velocity of the vibrating sphere. Figure 5f shows the post-FFT sensor response for varying frequency excitations of the dipole at 10 Hz, 35 Hz, 75 Hz, and 110 Hz. The results proved the sensor’s ability to respond to near-field perturbations in water, and can be used, for instance, in the underwater detection of objects especially in murky conditions with low visibility.

The processing methodology detailed in Figure 2 was thus able to successfully fabricate the 3D biomimetic sensor structure. In comparison with conventional ‘cleanroom’ approaches such as multilayer deposition and lithography, the proposed workflow is capable of monolithic fabrication of flexible electronics circuits, and can build both transducing (e.g. cilia-inspired pillars) and sensing (e.g. microchannels for piezoresistive materials such as GN) elements in the same step. Unlike direct 3D printing that is unable to print elastomeric polymers such as PDMS, our approach is not limited by the choice of polymer, and can, moreover, be used to fabricate multimaterial polymeric microstructures. With recent advances in 3D printing technology [58,59], feature sizes in the range of 1–30 µm are now achievable, making our proposed methodology ideal for batch fabrication of flexible microsensors of complex shapes. Finally, our method of 3D printing and molding is ideal for easy fabrication of arrays of cilia sensors similar to fish lateral lines.

## 4. Conclusions and Future Research

In this work, a novel processing methodology involving high-resolution metal 3D printing and polymer casting was developed to fabricate flexible, bioinspired, flow sensors. The PDMS sensor consisted of a cilium-inspired pillar and a cantilever with microchannels that housed a graphene nanoplatelet strain gauge. The gauge factor of the graphene-on-PDMS strain gauge was measured using cyclic tension-compression tests to be 37, an order of magnitude higher than comparable metal strain gauges. The bioinspired sensor was subjected to both tactile and flow stimuli, and displayed good sensitivity in all cases, showing a detection threshold of 12 µm for an oscillating tactile stimulus and 58 mm/s for an oscillatory flow stimulus in water. In conclusion, the developed fabrication method was successful for the fabrication of a soft polymer sensor and shows promise in batch fabrication of flexible electronics. Future work will focus on optimizing and miniaturizing the design of the sensor, optimizing the GN drop-casting procedure to ensure uniform and repeatable thin film characteristics, and fabricating cilia-inspired sensor arrays to mimic the fish lateral line. More generally, the approach presented in this work is part of a recent trend towards the utilization of 3D printing techniques for complex-shaped sensor fabrication. Recent developments in 3D printable technology—such as micron-scale printing resolutions and multi-material printing—can enable monolithic fabrication of biomimetic MEMS sensors with integrated piezoresisitive and/or piezoelectric sensing elements, thus obviating multi-step and expensive cleanroom fabrication processes. 

## Figures and Tables

**Table 1:** Comparison with gauge factor values in the literature.

Materials	Methods	Strain (%)	Gauge Factor	Reference
Graphene film on rubber	Spray coating	5	6–35	[43]
Graphene rosette strain gauge on PDMS film	Reactive ion etching, stamping	7.1	14	[38]
Graphene serpentine strain sensor on PDMS	Chemical vapor deposition, photolithography, spray coating	20	42.2	[40]
Graphene thin film on polyethylene terephthalate (PET) substrate	Spray deposition	1.5	10–100 (depending on graphene concentration)	[44]
Graphene nanoplatelets in microchannel on PDMS	PDMS casting inside 3D-printed mold, graphene ink drop-casting	±1.92	37	This work
